# COVID-19 pandemic reveals persistent disparities in nitrogen dioxide pollution

**DOI:** 10.1073/pnas.2022409118

**Published:** 2021-07-19

**Authors:** Gaige Hunter Kerr, Daniel L. Goldberg, Susan C. Anenberg

**Affiliations:** ^a^Department of Environmental and Occupational Health, Milken Institute School of Public Health, George Washington University, Washington, DC 20052;; ^b^Energy Systems Division, Argonne National Laboratory, Lemont, IL 60439

**Keywords:** nitrogen dioxide, air pollution, environmental justice, COVID-19, TROPOMI

## Abstract

The unequal spatial distribution of ambient nitrogen dioxide (${NO}_{2}$), an air pollutant related to traffic, leads to higher exposure for minority and low socioeconomic status communities. We exploit the unprecedented drop in urban activity during the COVID-19 pandemic and use high-resolution, remotely sensed ${NO}_{2}$ observations to investigate disparities in ${NO}_{2}$ levels across different demographic subgroups in the United States. We show that, prior to the pandemic, satellite-observed ${NO}_{2}$ levels in the least White census tracts of the United States were nearly triple the ${NO}_{2}$ levels in the most White tracts. During the pandemic, the largest lockdown-related ${NO}_{2}$ reductions occurred in urban neighborhoods that have 2.0 times more non-White residents and 2.1 times more Hispanic residents than neighborhoods with the smallest reductions. ${NO}_{2}$ reductions were likely driven by the greater density of highways and interstates in these racially and ethnically diverse areas. Although the largest reductions occurred in marginalized areas, the effect of lockdowns on racial, ethnic, and socioeconomic ${NO}_{2}$ disparities was mixed and, for many cities, nonsignificant. For example, the least White tracts still experienced ∼1.5 times higher ${NO}_{2}$ levels during the lockdowns than the most White tracts experienced prior to the pandemic. Future policies aimed at eliminating pollution disparities will need to look beyond reducing emissions from only passenger traffic and also consider other collocated sources of emissions such as heavy-duty vehicles.

## Significance

We leverage the unparalleled changes in human activity during COVID-19 and the unmatched capabilities of the TROPOspheric Monitoring Instrument to understand how lockdowns impact ambient nitrogen dioxide (${NO}_{2}$) pollution disparities in the United States. The least White communities experienced the largest ${NO}_{2}$ reductions during lockdowns; however, disparities between the least and most White communities are so large that the least White communities still faced higher ${NO}_{2}$ levels during lockdowns than the most White communities experienced prior to lockdowns, despite a ∼50% reduction in passenger vehicle traffic. Similar findings hold for ethnic, income, and educational attainment population subgroups. Future strategies to reduce ${NO}_{2}$ disparities will need to target emissions from heavy-duty vehicles.

Adverse air quality is an environmental justice issue, as it disproportionately affects marginalized and disenfranchised populations around the world (1–4). Growing evidence suggests that these populations experience more air pollution than is caused by their consumption (5–7). Within the United States, disparities in exposure are persistent, despite successful regulatory measures that have reduced pollution (8, 9). Nitrogen dioxide (${NO}_{2}$) is a short-lived trace gas formed shortly after fossil fuel combustion and regulated by the National Ambient Air Quality Standards under the Clean Air Act. Exposure to ${NO}_{2}$ is associated with a range of respiratory diseases and premature mortality (10–12). ${NO}_{2}$ is also a precursor to other pollutants such as ozone and particulate matter (13). Major sources of anthropogenic ${NO}_{2}$, such as roadways and industrial facilities, are often located within or nearby marginalized and disenfranchised communities (14, 15), and disparities in ${NO}_{2}$ exposure across demographic subgroups have been the focus of several recent studies (4, 8, 16–18).

In early 2020, governments around the world imposed lockdowns and shelter-in-place orders in response to the spread of COVID-19. The earliest government-mandated lockdowns in the United States began in California on 19 March 2020, and many states followed suit in the following days. Changes in mobility patterns indicate that self-imposed social distancing practices were underway days to weeks before the formal announcement of lockdowns (19). Lockdowns led to sharp reductions in surface-level ${NO}_{2}$ (20–23) and tropospheric column ${NO}_{2}$ measured from satellite instruments (21, 24–27) over the United States, China, and Europe. According to government-reported inventories, roughly 60% of anthropogenic emissions of nitrogen oxides (${NO}_{x}\equiv$ NO + ${NO}_{2}$) in the United States in 2010 were emitted by on-road vehicles (28), and up to 80% of ambient ${NO}_{2}$ in urban areas can be linked to traffic emissions (29, 30). As such, ${NO}_{2}$ is often used as a marker for road traffic in urban areas. Multiple lines of evidence such as seismic quieting and reduced mobility via location-based services point to changes in traffic-related emissions as the main driver of reductions in ${NO}_{2}$ pollution during lockdowns, due to the large proportion of the population working from home (21, 23, 31, 32).

Here we exploit the unprecedented changes in human activity unique to the COVID-19 lockdowns and remotely sensed ${NO}_{2}$ columns with extraordinary spatial resolution and coverage to understand inequalities in the distribution of ${NO}_{2}$ pollution for different racial, ethnic, and socioeconomic subgroups in the United States. Specifically, we address the following: Which demographic subgroups received the largest ${NO}_{2}$ reductions? Did the lockdowns grow or shrink the perennial disparities in ${NO}_{2}$ pollution across different demographic subgroups? Although the lockdowns are economically unsustainable, how can they advance environmental justice and equity by informing long-term policies to reduce ${NO}_{2}$ disparities and the associated public health damages?

## Results

**Fig. 1.**
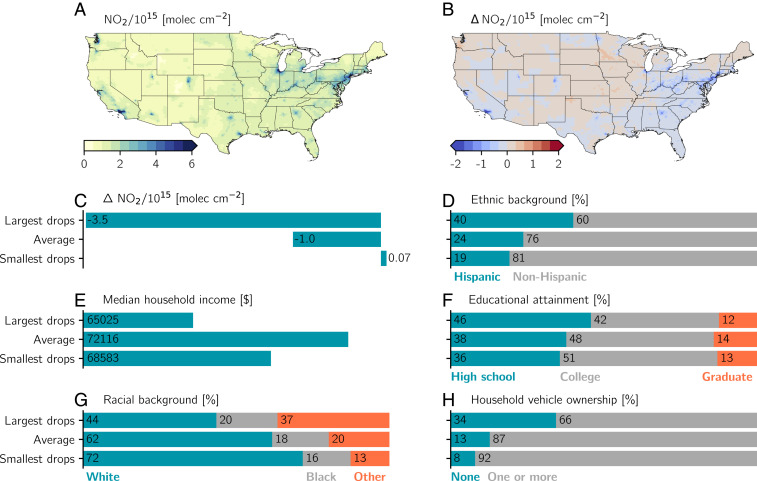
Spatial distribution of ${NO}_{2}$ columns during the baseline and COVID-19 lockdown periods and apportionment of drops among different demographic subgroups. (*A*) Census tract average baseline ${NO}_{2}$ (13 March to 13 June 2019). (*B*) Absolute difference between lockdown (13 March to 13 June 2020) and baseline ${NO}_{2}$ ($\Delta \;{NO}_{2}$), where $\Delta \;{NO}_{2}<0$ corresponds to ${NO}_{2}$ drops during lockdowns. (*C*–*H*) Demographic data averaged over urban tracts with the largest drops ($\Delta \;{NO}_{2}$ in first decile), all urban tracts, and urban tracts with the smallest drops ($\Delta \;{NO}_{2}$ in the tenth decile). “Other” in *G* includes American Indian or Alaska Native, Asian, Native Hawaiian or other Pacific Islander, two or more races, and some other race. The census-designated concept of race differs from ethnicity, and the percentage of White residents in *G* includes individuals with Hispanic origin or descent.

Previous studies examining satellite-derived ${NO}_{2}$ found the highest levels in urban areas (33–35), and we find that these areas clearly stand out as ${NO}_{2}$ hotspots during our baseline period (Fig. 1*A*). ${NO}_{2}$ column densities averaged over all urban areas are ∼2 times higher than over rural areas during the baseline period. Absolute differences in ${NO}_{2}$ between the baseline and lockdown periods (“drops”) show sharp decreases over virtually all major metropolitan regions (Fig. 1*B*). The use of only spring 2019 for our baseline period stems from the short data record offered by the Tropospheric Monitoring Instrument (TROPOMI), and the slight increases in ${NO}_{2}$ in parts of the Great Plains and Midwest during lockdowns ($<0.5\times 10^{15}$ molecules per square centimeter) could reflect differences in natural (e.g., soil, lightning, stratospheric ${NO}_{x}$) or anthropogenic sources of ${NO}_{2}$ between the baseline and lockdown periods. Demetillo et al. (4) found that TROPOMI is capable of resolving ${NO}_{2}$ differences between census tracts in the Houston area, and our nationwide comparison of TROPOMI ${NO}_{2}$ with surface-level observations reveals TROPOMI’s utility as a tool to understand ${NO}_{2}$ variability (*SI Appendix*, Text S1 and Fig. S1). The 3-mo baseline and lockdown periods used in this study have sufficient length to account for the influence of meteorological variability on ${NO}_{2}$, and ${NO}_{2}$ disparities found using a 3-mo period closely resemble disparities calculated with longer timeframes (*SI Appendix*, Fig. S2). Given that the largest lockdown-related changes in ${NO}_{2}$ occur in urban areas and to avoid urban–rural demographic gradients, we primarily focus on urban ${NO}_{2}$ changes and how these changes impact different demographic subgroups in urban areas.

The largest urban ${NO}_{2}$ drops occur in census tracts that are more non-White and Hispanic, have lower median household income, and have a higher proportion of their population without a vehicle or a postsecondary education compared with tracts with the smallest drops (Fig. 1 *D*–*H*). In tracts with the largest drops, there are ∼2.0 times more non-White residents and ∼2.1 times more Hispanic residents than in tracts with the smallest drops (Fig. 1 *D* and *G*). The differences in the “Other” category between tracts with largest and smallest drops (Fig. 1*G*) reflect differences in the Asian population (5% in tracts with the smallest drops; 14% in tracts with the largest drops) and the proportion of the population that does not identify as one of the census-designed racial categories (4% in tracts with smallest drops; 19% in tracts with the largest drops). These results for urban tracts also hold in all (urban and rural) tracts and rural tracts, despite the different demographic composition (compare Fig. 1 and *SI Appendix*, Fig. S3). Differences in distributions of demographic variables between tracts with the largest versus smallest drops in Fig. 1 *C*–*H* are all statistically significant.

Communities with lower income and educational attainment and a large proportion of racial and ethnic minorities have faced higher levels of ${NO}_{2}$ and other pollutants for decades (3, 8, 9, 16, 36), and we find that these communities experienced the largest drops in ${NO}_{2}$ pollution during COVID-19 lockdowns. However, Fig. 1 does not indicate how lockdown-related ${NO}_{2}$ drops grew or shrunk disparities, and we next examine disparities in baseline and lockdown ${NO}_{2}$ in the most marginalized versus least marginalized census tracts in the United States.

**Fig. 2.**
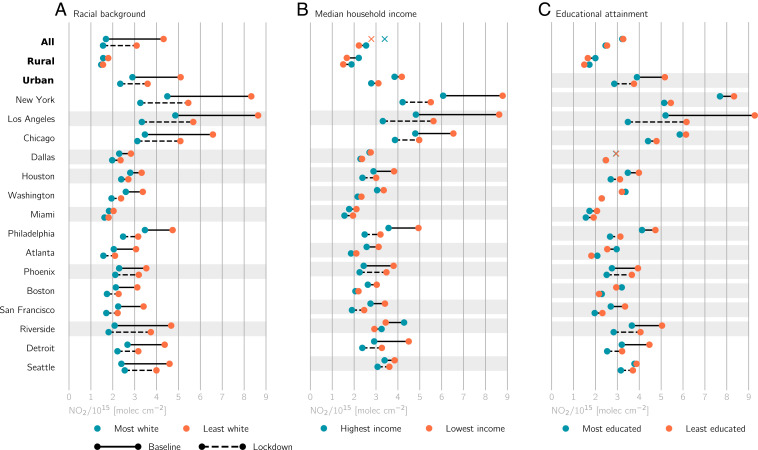
Disparities in baseline and lockdown ${NO}_{2}$ columns for different (*A*) racial, (*B*) median household income, and (*C*) educational attainment population subgroups. Disparities are shown for three conglomerations (all, urban, and rural census tracts), and urban tracts are further separated into the 15 largest MSAs in the United States. For each conglomeration or MSA, demographic subgroups are determined using the 10th and 90th percentiles as thresholds. ${NO}_{2}$ levels are thereafter averaged over tracts within these subgroups. If the difference in subgroup ${NO}_{2}$ distributions for a particular demographic variable and time period is not statistically significant, mean ${NO}_{2}$ levels are denoted with an “X” and no connector lines. Conglomerations or MSAs with no significant change in ${NO}_{2}$ disparities between the baseline and lockdown periods are shaded in gray.

In the baseline and lockdown periods, neighborhoods with lower income and educational attainment and those with a larger proportion of minority residents consistently face higher levels of ${NO}_{2}$ among all urban tracts across the United States and in nearly all of the 15 largest metropolitan statistical areas (MSAs) in the United States (Fig. 2 and *SI Appendix*, Fig. S4). There are some cases in which the most marginalized tracts do not experience the highest ${NO}_{2}$ levels. For example, rural tracts with the highest income and educational attainment have higher ${NO}_{2}$ levels than tracts with the lowest income or educational attainment (Fig. 2 *B* and *C*), and similar findings hold for specific MSAs (e.g., Riverside in Fig. 2*B*, Atlanta in Fig. 2*C*). Moreover, there are no significant differences in ${NO}_{2}$ distributions for tracts with the highest versus lowest income during the baseline period (Fig. 2*B*).

When considering all census tracts (both urban and rural), the most pronounced disparities, defined as the ratio of mean ${NO}_{2}$ for the marginalized subgroup to the nonmarginalized subgroup, are on the basis of race and ethnicity. The least White tracts and most Hispanic tracts have 2.6 and 2.2 times greater baseline ${NO}_{2}$ levels than the most White and least Hispanic tracts, respectively (Fig. 2*A* and *SI Appendix*, Figs. S4*A* and S5*G*). These disparities persist when examining individual MSAs in the United States. For example, baseline ${NO}_{2}$ in tracts with the lowest median household income in New York and Los Angeles is 1.4 and 1.8 times higher, respectively, than in tracts with the highest income (Fig. 2*B* and *SI Appendix*, Fig. S4*B*).

The unprecedented change in human activity during COVID-19 lockdowns led to mixed impacts on relative ${NO}_{2}$ disparities across different population subgroups, depending on the demographic variable and MSA considered (Fig. 2 and *SI Appendix*, Fig. S4). Racial ${NO}_{2}$ disparities for all census tracts significantly decreased from 2.6 to 2.0 during lockdowns, and a majority of the featured MSAs experienced significant reductions in their racial disparities (Fig. 2*A* and *SI Appendix*, Fig. S4*A*). Disparities for other demographic variables, however, were less affected by lockdowns. For example, a majority of MSAs had no significant reduction in disparities for different levels of income and educational attainment (Fig. 2 *B* and *C* and *SI Appendix*, Fig. S4 *B* and *C*). Understanding inconsistencies in the exact magnitude of ${NO}_{2}$ drops across MSAs for different population subgroups is beyond the scope of this study but could stem from varying stringencies of or adherence to lockdown measures.

Although urban areas experienced broad drops in ${NO}_{2}$ during lockdowns, with the largest drops occurring in marginalized neighborhoods (Fig. 1 *C*–*H*), ${NO}_{2}$ disparities in the baseline period were so large that even significant reductions in disparities did not generally bring lockdown ${NO}_{2}$ levels for marginalized neighborhoods to the levels experienced by nonmarginalized neighborhoods during the baseline period (Fig. 2). As an example, despite the unprecedented drop in human activity during the COVID-19 pandemic, ${NO}_{2}$ levels in the least White neighborhoods in New York and Chicago were $∼1\times 10^{15}$ and $∼2\times 10^{15}$ molecules per square centimeter higher, respectively, during lockdowns than levels in the most White neighborhoods during the baseline period. Houston, Washington, Philadelphia, and San Francisco are notable exceptions to this result, and ${NO}_{2}$ levels for the least White tracts during lockdowns fell below ${NO}_{2}$ levels for the most White tracts during the baseline period in these cities. We observe similar results for population subgroups based on ethnicity, income, and educational attainment (Fig. 2 and *SI Appendix*, Figs. S4 and S5).

**Fig. 3.**
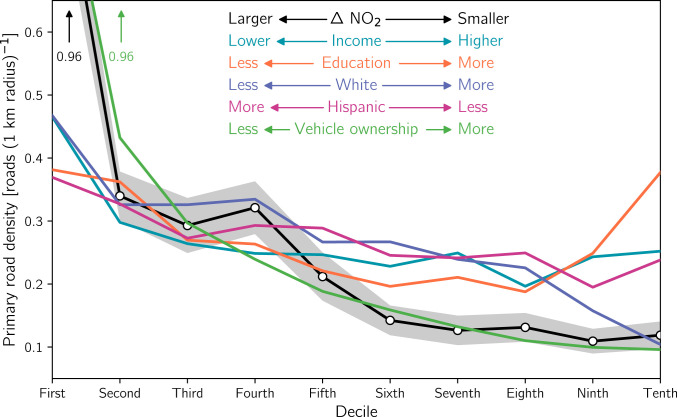
The relationship of road density with urban lockdown-related drops in ${NO}_{2}$ columns and demographic variables. Road density is calculated as the number of primary road segments within a 1-km radius of tracts’ centroids for each decile of demographic variables. The colored legend indicates the directionality of each demographic variable. As an example, the density corresponding to the lowest decile of the “White” curve represents the road density in urban tracts that are the least White (i.e., in the first decile of the percentage of their population that is White). Shading for the $\Delta {NO}_{2}$ curve illustrate the 90% CI.

Within urban areas, we find that the magnitude of ${NO}_{2}$ drops is tightly coupled to the density of nearby primary roads (highways and interstates). The density of primary roads in urban tracts with the largest ${NO}_{2}$ drops (i.e., tracts in the first decile) is 9.5 times greater than in urban tracts with the smallest ${NO}_{2}$ drops (i.e., tenth decile) (Fig. 3). The racial, ethnic, income, and educational compositions of tracts are also closely related to primary road density. Urban tracts with lower income and vehicle ownership and a larger percentage of racial and ethnic minorities are located near a higher density of primary roads (Fig. 3). The difference in primary road density on the basis of vehicle ownership is especially stark: Tracts with the lowest vehicle ownership have a ∼9.5 times higher primary road density than tracts with the highest ownership. Similarly, the least White tracts have a primary road density ∼4.5 times higher than the most White tracts. Educational attainment is the only demographic variable considered in this study that exhibits a different relationship with primary road density, and we observe a U-shaped relationship between these variables (Fig. 3).

**Fig. 4.**
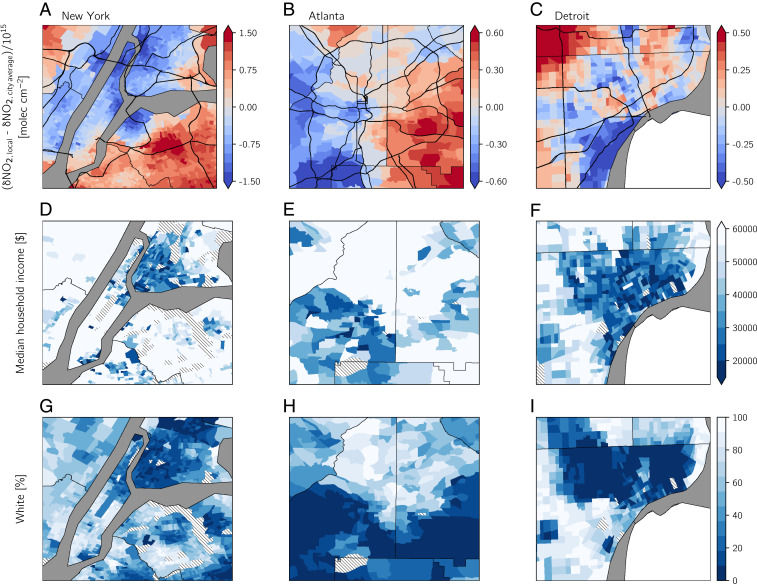
Case studies of lockdown ${NO}_{2}$ drops, income, and race for (*A*, *D*, and *G*) New York, (*B*, *E*, and *H*) Atlanta, and (*C*, *F*, and *I*) Detroit. (*A*–*C*) $\Delta \;{NO}_{2,\text{local}}$ is calculated from oversampled TROPOMI data as the difference between $\Delta \;{NO}_{2}$ and the city average $\Delta \;{NO}_{2}$ to highlight neighborhoods with larger drops (i.e., negative values) and smaller drops (i.e., positive values) compared with the city-averaged drops. Primary roads are shown in thick black lines. (*D*–*F*) Median household income and (*G*–*I*) percentage of the population that is White. Tracts in *D*–*I* that are employment centers, airports, parks, or forests and therefore report no demographic data are denoted with hatching.

To better understand the impact of the lockdowns on ${NO}_{2}$ disparities, we consider case studies of individual cities: New York, Detroit, and Atlanta (Fig. 4). Among individual neighborhoods in each of these cities, the magnitude of ${NO}_{2}$ drops varies up to 50% above and below the citywide average (Fig. 4 *A*–*C*). The portions of New York, Atlanta, and Detroit that received the largest drops tend to have lower median household income and a high percentage of non-White residents (Fig. 4 *D*–*I*). Although the sharp decrease in passenger vehicle emissions (21, 23, 37) is the primary factor in explaining the large-scale ${NO}_{2}$ drops, examining drops on smaller neighborhood scales in New York, Atlanta, Detroit (Fig. 4), or other MSAs suggests that other sectors may contribute to the ${NO}_{2}$ drops, in addition to on-road activity. In New York, the largest drops are concentrated in Harlem and the South Bronx (Fig. 4*A*), where the high concentration of major highways and industrial facilities has been linked to disproportionate exposure to air pollution (38). The largest drops in Atlanta occur in the southwestern part of the city, where median household income generally is <$30,000 and the percentage of Black residents in each tract is nearly 100. Hartsfield-Jackson International Airport and several major highways are located in this part of Atlanta (Fig. 4*B*). The airport reported a ∼50% decrease in the daily number of flights during lockdowns (39). Therefore, both on-road and aviation emissions may be responsible for the disparities in ${NO}_{2}$ levels in Atlanta. The largest drops in Detroit are concentrated on the west shores of the Detroit River; Interstates 75 and 94 and the Ambassador Bridge, one of the busiest US–Canada border crossings, transect this part of Detroit (Fig. 4*C*) (40). Although these Detroit neighborhoods are not predominantly non-White (Fig. 4*F*), they are home to a large Hispanic population with low median household income (Fig. 4*I*).

## Discussion

Neighborhoods with a large proportion of racial and ethnic minorities, lower income, and lower educational attainment saw the greatest decreases in ${NO}_{2}$ pollution during the COVID-19 lockdowns. Although lockdowns were lauded as a temporary glimpse of the potential for cleaner urban air, ${NO}_{2}$ disparities persisted during this global natural experiment. For many cities, there were no significant changes in ${NO}_{2}$ disparities during the lockdowns, and marginalized communities faced higher ${NO}_{2}$ levels during the lockdowns than nonmarginalized communities experienced prior to the lockdowns. Our findings build on Demetillo et al. (4), who similarly used TROPOMI to understand environmental justice in Houston and inform drivers of inequality, and are consistent with contemporaneous studies that have analyzed long-term trends in ${NO}_{2}$ and other air pollutants and found that, despite widespread decreases in pollution, the most exposed demographic subgroups in the 1980s and 1990s remain the most exposed in the present day (8, 9).

Sources of urban ${NO}_{2}$ such as railroads, ports, airports, or industrial facilities are not disproportionately located in marginalized neighborhoods, do not contribute in a major way to total urban ${NO}_{x}$ emissions, or were not largely affected by the pandemic (*SI Appendix*, Text S1 and Figs. S6–S8). The location of primary roads, however, is heavily skewed toward marginalized neighborhoods (Fig. 3), and on-road emissions from light- and heavy-duty vehicles represent a sizable contribution (∼40 to 50%) to urban ${NO}_{x}$ emissions (*SI Appendix*, Fig. S7). The collocation of primary roads with poor, minority communities is not happenstance but a consequence of the Eisenhower-era federal highway program, which often deliberately routed highways through these poor, minority neighborhoods (8, 15, 41, 42). While passenger vehicle traffic experienced a precipitous decline during the pandemic (21, 23, 37), heavy-duty trucking largely continued unabated (*SI Appendix*, Fig. S8). Together, these findings indicate that heavy-duty trucking plays a major role in explaining persistent disparities of ${NO}_{2}$ pollution among demographic subgroups. As was previously pointed out with the case studies of New York, Atlanta, and Detroit (Fig. 4), ${NO}_{2}$ sources beyond on-road transportation may be important to understand ${NO}_{2}$ disparities locally, but the small contribution of these other sources to total urban ${NO}_{x}$, their small or inconsistent changes during lockdowns, and their point source nature suggest that they are unlikely to explain the nationwide urban ${NO}_{2}$ disparities detailed herein.

Interestingly, urban tracts with the lowest vehicle ownership have both the highest density of nearby primary roads and the largest drops in ${NO}_{2}$ (Figs. 1*H* and 3). This result suggests that these communities may breathe more traffic-related ${NO}_{2}$ pollution than they produce. This is indeed the case for particulate matter pollution: Recent work found that particulate matter exposure is disproportionately caused by wealthy, non-Hispanic White communities, while poor, Black, and Hispanic communities face higher exposure than is caused by their own consumption (6, 7).

Preliminary research suggests that high levels of ${NO}_{2}$ pollution contribute to underlying health conditions that lead to increased COVID-19 fatality rates (43). Therefore, the decrease in ${NO}_{2}$ in diverse communities with low income or educational attainment could decrease population susceptibility to COVID-19. This result is especially important as these communities have increased risk for COVID-19 and higher hospitalization rates (44). Since short-term ${NO}_{2}$ exposure is associated with respiratory disease (45, 46), the temporary ${NO}_{2}$ drops may have also reduced acute respiratory health outcomes, but the actual health effects of ${NO}_{2}$ drops during the pandemic are difficult to tease out since the degree to which people sought health care was also affected by the pandemic. These findings are especially relevant for marginalized neighborhoods in cities (e.g., New York, Atlanta, and Detroit; Fig. 4) that have been long plagued by high rates of asthma and other respiratory diseases due, in part, to their proximity to on-road and point source ${NO}_{x}$ emissions (38, 40).

We have considered singular demographic variables and their relationship with baseline and lockdown ${NO}_{2}$. The case studies in Fig. 4 hint that the intersectionality between race and poverty may be associated with even more pronounced lockdown-related drops in ${NO}_{2}$ pollution. Although the vast majority of tracts in the southern half of Atlanta have a majority non-White population (Fig. 4*H*), the largest drops occur in tracts that are both majority non-White and low income (Fig. 4 *B*, *E*, and *H*). Clark et al. (17) and Demetillo et al. (4) examined ${NO}_{2}$ exposure in neighborhoods where poverty and racial and ethnic identities intersect and found a disproportionate share of ${NO}_{2}$ pollution for neighborhoods with these intersecting identities. Assessing other forms of intersectionality and their relationship with air pollution exposure is a key area for future research.

Recent work demonstrates that satellite-observed ${NO}_{2}$ is a powerful proxy for ground-level ${NO}_{2}$ gradients (47), and TROPOMI, in particular, provides significant advances over predecessor instruments, on account of its unprecedented spatial resolution (48). We tested whether TROPOMI has consistent spatial patterns with surface-level observations during the baseline period and found good agreement (*SI Appendix*, Fig. S1*A*). TROPOMI’s correlation with surface-level monitors (*SI Appendix*, Text S1 and Fig. S1*A*) is a dramatic improvement over predecessor instruments (49). Moreover, the ratios of 24-h average ${NO}_{2}$ to ${NO}_{2}$ near the time of satellite overpass are also similar between least and most polluted sites (*SI Appendix*, Fig. S1*B*). We note, however, that satellite-derived ${NO}_{2}$ tends to underestimate ${NO}_{2}$ in highly polluted urban regions, on account of satellite footprint resolution (50). This underestimation, coupled with the fact that marginalized communities tend to live closer to potent ${NO}_{2}$ sources such as highways (Fig. 3) that cannot be resolved given TROPOMI’s resolution, suggests that our current methodology may underestimate the magnitude of disparities and lockdown-related changes.

Our results are neither an artifact of how we defined demographic subgroups (*SI Appendix*, Fig. S5) nor the time period over which we characterize disparities, although the precise absolute ${NO}_{2}$ levels and magnitude of disparities change with the start dates and length of the periods (*SI Appendix*, Figs. S2 and S9). We encourage future work using surface-level ${NO}_{2}$ concentrations to better understand exposure across demographic subgroups during lockdowns. Current surface-level observational networks are inadequate for doing so, due to their sparse and uneven distribution (51), but surface concentrations of ${NO}_{2}$ observed from networks of low-cost sensors (52) or inferred using land-use regression models (53) and chemical transport models (34, 54) may prove useful. Future work might also examine how lockdown-related changes in other air pollutants such as ozone and particulate matter, whose changes during lockdowns do not exhibit the same spatial patterns as ${NO}_{2}$ (22, 23, 55), impact disparities.

## Conclusions

This study provides a unique look at air pollution disparities in the United States, leveraging the confluence of unparalleled changes in human activity during COVID-19 lockdowns and the unmatched spatial coverage and resolution of air quality surveillance from the TROPOMI satellite instrument. Lockdowns decreased tropospheric column abundances of ${NO}_{2}$ across the vast majority of urban areas. However, drops in ${NO}_{2}$ pollution were uneven within these urban areas, and the largest drops occurred in communities with a high proportion of racial and ethnic minorities and lower educational attainment and income. Our results reveal that, despite the decreases in ${NO}_{2}$ pollution during lockdowns, racial, ethnic, and socioeconomic ${NO}_{2}$ disparities persisted, and marginalized communities continued to face higher levels of ${NO}_{2}$ during the lockdowns than nonmarginalized communities experienced prior to the pandemic. As passenger vehicles represent a large source of urban ${NO}_{x}$ emissions, the proximity of marginalized neighborhoods to a high density of major roadways is likely the key determinant in explaining lockdown-related drops in ${NO}_{2}$.

Our results offer insight into policies aimed at reducing or eliminating ethnoracial and socioeconomic ${NO}_{2}$ disparities. The COVID-19 lockdowns showed that a dramatic drop in ${NO}_{x}$ emissions mainly from the passenger vehicle sector narrowed ${NO}_{2}$ disparities only modestly and not consistently across major US cities. Heavy-duty diesel vehicles, on the other hand, maintained more or less the same activity levels during the COVID-19 lockdowns, continue to be a major contributor to urban ${NO}_{x}$ emissions, and use highways and interstates disproportionately located in marginalized communities. While decreasing ${NO}_{x}$ emissions from passenger vehicles, airports, railways, ports, and industry would broadly reduce ${NO}_{2}$ levels and is relevant for disparities in some cities, targeting ${NO}_{x}$ emissions from heavy-duty diesel vehicles is likely the most effective strategy for reducing disparities across cities nationwide. Future studies and policy strategies should therefore examine how targeting heavy-duty diesel traffic can address inequity in exposure while maximizing health benefits.

## Materials and Methods

### Remotely Sensed ${NO}_{2}$.

We obtain retrievals of the tropospheric ${NO}_{2}$ column from the TROPOMI aboard the Sentinel-5 Precursor (S5P) satellite. S5P is a nadir-viewing satellite in a sun-synchronous, low-Earth orbit that achieves near-global daily coverage with a local overpass time of ∼1,330 h (56). TROPOMI provides ${NO}_{2}$ measurements at an unprecedented spatial resolution of 5×3.5 km (7×3.5 km prior to 6 August 2019) (57). We use level 2 data and only consider pixels with a quality assurance value of >0.75. The change in satellite resolution occurring in August 2019 as well as intrinsic limitations stemming from the retrieval process and satellite footprint likely lead to an underestimation of ${NO}_{2}$ levels in urban areas and potentially the ${NO}_{2}$ change during lockdowns (47, 50). TROPOMI data are thereafter oversampled by regridding to a standard grid with a resolution of 0.01° latitude ×0.01° longitude (∼1×1 km) and averaged over two time periods: a baseline period (13 March to 13 June 2019) and a lockdown period (13 March to 13 June 2020). Regridded data are publicly available at Figshare (https://figshare.com/s/75a00608f3faedc4bca7).

Comparing the same time period across different years is commonplace in satellite studies investigating changes in ${NO}_{x}$ and other trace gases, and averaging over 3-mo timeframes smooths natural ${NO}_{2}$ variations that arise from differences in meteorology and sun angle, which are especially relevant during boreal spring (26) (*SI Appendix*, Fig. S2). This temporal averaging also removes most of the random error in the TROPOMI single-pixel uncertainties, which can be 40 to 60% of the tropospheric column abundances (24).

### Sociodemographic Data.

Demographic information is derived from the American Community Survey (ACS) conducted by the US Census Bureau and maintained by the National Historical Geographic Information System (58). Data are publicly available at https://www.nhgis.org. We extract 2014–2018 5-y estimates on race, Hispanic or Latino origin (henceforth “ethnicity”), educational attainment, median household income, and vehicle availability for the 72,538 census tracts in the contiguous United States. To minimize the number of different categorical variables presented in this study, we combine racial groups into three categories: White, Black (includes Black and African American), and Other (includes American Indian or Alaska Native, Asian, Native Hawaiian or Other Pacific Islander, two or more races, and some other race). Similarly, we form three different levels for educational attainment: high school (includes no high school diploma, regular high school diploma, and GED or alternative credentials), college (includes some college without a degree, associate’s degree, and bachelor’s degree), and graduate (includes master’s degree, professional school degree, and doctorate degree).

### Methods

We harmonize the regridded TROPOMI ${NO}_{2}$ measurements with tract-level ACS demographics by determining the geographic boundaries of each tract and thereafter calculating a simple arithmetic average over all TROPOMI grid cells within the tract for the baseline and lockdown periods. While the area of most census tracts is much larger than the ∼1×1 km TROPOMI grid cells (*SI Appendix*, Fig. S10), approximately 8% of tracts lack a colocated grid cell, due to their small size (or irregular geometry). For example, the median area of census tracts in New York is $0.7\;{km}^{2}$ (*SI Appendix*, Fig. S10). For these small tracts, we employ inverse distance weighting interpolation to calculate the ${NO}_{2}$ levels at their centroids using ${NO}_{2}$ levels in the eight neighboring grid cells. This approach may smooth over the fine-scale ${NO}_{2}$ gradients present in very small tracts and potentially underestimate the impacts of ${NO}_{x}$ emissions (4). Tracts are classified as either rural or urban based on the census-designed rurality level from the last decadal census in 2010. Urban census tracts lie within the boundaries of an incorporated or census-designed place with >2,500 residents, and rural tracts are located outside these boundaries. Therefore, suburban areas on the periphery of cities with >2,500 residents are classified as “urban” in this study. We further stratify the tracts into metropolitan-level subsets for the 15 largest MSAs in the United States: New York City–Newark–Jersey City, NY–NJ–PA; Los Angeles–Long Beach–Anaheim, CA; Chicago–Naperville–Elgin, IL–IN–WI; Dallas–Fort Worth–Arlington, TX; Houston–The Woodlands–Sugar Land, TX; Washington–Arlington–Alexandria, DC–VA–MD–WV; Miami–Fort Lauderdale–Pompano Beach, FL; Philadelphia–Camden–Wilmington, PA–NJ–DE–MD; Atlanta–Sandy Springs–Alpharetta, GA; Phoenix–Mesa–Chandler, AZ; Boston–Cambridge–Newton, MA–NH; San Francisco–Oakland–Berkeley, CA; Riverside–San Bernardino–Ontario, CA; Detroit–Warren–Dearborn, MI; and Seattle–Tacoma–Bellevue, WA. For brevity, we refer to these MSAs by their colloquial names (e.g., Los Angeles, rather than Los Angeles–Long Beach–Anaheim, CA) when discussing them.

We calculate the density of nearby primary roadways for each census tract as a proxy for exposure to traffic-related ${NO}_{2}$ pollution. Primary roads are generally divided, limited-access highways within the Interstate Highway System or under state management, and their locations are determined from the US Census Bureau’s TIGER/Line geospatial database. Specifically, we determine density as the number of primary road segments within 1 km of a tract’s centroid. We choose 1 km as our threshold for “nearby,” as ${NO}_{2}$ concentrations decrease up to ∼50% within 0.5 km to 2 km from major roadways (4, 53). Other means of quantifying traffic exist (e.g., length of roadway within a specified distance, traffic within buffer zones, sum of distance traveled) (59), but our approach allows for consistent use of geospatial data from the US Census Bureau.

We partition census tracts by extreme values of their change in ${NO}_{2}$ ($\Delta \;{NO}_{2}$) or demographic variables using the first decile (0 to 10th percentile) and tenth decile (90th to 100th percentile). As examples, tracts classified as “most White” or “highest income” have a White population fraction or median household income which falls in the tenth decile. Similarly, $\Delta \;{NO}_{2}$ in tracts with the “largest drops” (i.e., the largest decrease in ${NO}_{2}$ during lockdowns) falls in the first decile. Decile thresholds are defined separately for all, urban, and rural census tracts and for different MSAs to account for urban–rural gradients and differences among MSAs. We note that, when this approach is applied to all (urban and rural) census tracts, a broad distribution of tracts is selected, not just tracts from a certain geographic region; for example, the ∼7,200 tracts classified as “most White” for all urban and rural census tracts represent tracts from all 48 states in the contiguous United States and Washington, DC. Our results are not sensitive to the use of the first and tenth deciles, and we have tested the upper and lower vigintiles, quintiles, and quartiles and obtained similar results (*SI Appendix*, Fig. S5). The use of percentiles rather than absolute thresholds yields a consistent sample size for the upper and lower extrema and also avoids defining absolute thresholds for different variables.

We applied the two-sample Kolmogorov–Smirnov (KS) test to determine whether distributions of demographic variables in tracts with the largest and smallest ${NO}_{2}$ drops (Fig. 1 *C*–*H*) and tract-averaged ${NO}_{2}$ for the upper and lower extrema of demographic variables (Fig. 2) are drawn from the same distribution (*SI Appendix*, Fig. S11). If the p value corresponding to the KS test statistic is less than α=0.05, we declare that there are significant differences in the distributions. We also assess whether the ${NO}_{2}$ disparities shown in Fig. 2 undergo significant changes between the baseline and lockdown periods, using a two-sample z test. To meet the normality assumption of the z test, we log-transform the skewed ${NO}_{2}$ distributions prior to computing the test statistic. Changes in baseline versus lockdown disparities are classified as significant when the absolute value of the test statistic is larger than 1.96, the critical value for a 95% level of confidence (p<0.05). We note that this approach to assess the significance of changes in disparities agrees well with other methods, such as examining whether 95% confidence levels of the baseline and lockdown disparities overlap (compare Fig. 2 and *SI Appendix*, Fig. S4).

The start date of the baseline and lockdowns periods used in this study (13 March) corresponds to the date of national emergency declaration in the United States and the beginning of a pronounced decrease in mobility patterns in 2020 (19). We test whether the overall racial, ethnic, income, and educational disparities hold for other periods and find that the disparities among different demographic subgroups persist regardless of the start date or length of the baseline period (*SI Appendix*, Figs. S2 and S9). We are inherently limited by the short TROPOMI data record, and interannual variability could play a role in modulating the magnitude of disparities in ${NO}_{2}$ levels. Testing this possibility is important as more TROPOMI data become available.

## Supplementary Material

Supplementary File

## Data Availability

All study data are included in the article and *SI Appendix*.
