# Supplementary material for: COVID-19 pandemic reveals persistent disparities in nitrogen dioxide pollution
Source: Proc Natl Acad Sci U S A. 2021 Jul 19;118(30):e2022409118. doi: 10.1073/pnas.2022409118 (PMC8325165; doi:10.1073/pnas.2022409118)
Supplement: Supplementary File [file pnas.2022409118.sapp.pdf]

PNAS
www.pnas.org

Supplementary Information for

COVID-19 pandemic reveals persistent disparities in nitrogen dioxide pollution

Gaige Hunter Kerr, Daniel L. Goldberg and Susan C. Anenberg

Corresponding Author G. H. Kerr
E-mail: gaigekerr@gwu.edu

This PDF file includes:

Supplementary text
Figs. S1 to S11
SI References

Supporting Information Text

Remotely-sensed versus surface-level NO_2

We compare tropospheric column NO_2 from TROPOMI with ground-based observations from the Environmental Protection Agency's Air Quality System (AQS) (1) to test whether TROPOMI can provide an accurate characterization of differences in surface-level NO_2 during the baseline period (13 March - 13 June 2019). There are 439 AQS monitors in the contiguous U.S. with observations during the baseline period, and we average hourly observations over the entire baseline period at each of these sites and compare them with TROPOMI retrievals at the collocated grid cell to each site.

We find that 71 of the 439 monitors are located near ($<$ 20 meters) roads (2). These sites generally have observed surface-level $NO_2 > 10$ ppbv despite relatively low columnar amounts from TROPOMI (Figure S1). We do not expect TROPOMI to capture the steep gradients of NO_2 near roadways on account of the differences in scale between the footprint of the satellite and point-based observations. When we consider only AQS monitors that are not located near roads, we find good agreement between these surface-level observations and TROPOMI (Figure S1a). We also find a similar ratio of NO_2 averaged over the 24-hour diurnal cycle to NO_2 near the time of satellite overpass at sites that are classified as the most and least polluted (Figure S1b). Additional factors such as instrument error (for both TROPOMI and AQS) and clear sky biases may contribute to deviations from a perfect linear relationship between the space-based and surface-level observations (3–5); however, the findings of this analysis lend credibility to our reliance on TROPOMI to characterize disparities in NO_2 at earth's surface.

Identifying drivers of COVID-19 NO_2 changes

To help understand which NO_2 sources drive lockdown-related drops, we consider several other important sources of NO_x emissions beyond on-road traffic: ports (6), railroads (7), airports (8), and stationary industrial sources with continuous emissions monitoring systems (CEMS) measuring effluent emission levels from flue-gas stacks (9). Using the geographic locations of each of these different NO_x sources, we calculate the density of each source for different demographic subgroups in a similar fashion to our analysis of roadways in Figure 3 of the main text.

The locations of ports and airports in urban areas are not skewed towards neighborhoods with low income, low educational attainment, or a large share of minorities (Figure S6a-c). We also have examined whether industrial sources with the highest NO_x emissions are located in marginalized neighborhoods, but we find that this is not the case (Figure S6b). The exception to this finding is that industrial stationary sources with the highest NO_x emissions have a tendency to be located in in census tracts with lower educational attainment and income (Figure S6b), but this relationship is far weaker than the relationship of educational attainment and income with roadways (Figure 3 in the main text). This result that stationary industrial sources are not preferentially located in marginalized neighborhoods is surprising, and we note that some small facilities may not require CEMS and are thus not included in this analysis. Additionally, we have not examined the locations of other stationary facilities which emit dozens of other hazardous air pollutants. While these other facilities might not impact the NO_2 disparities investigated herein, they still could be relevant for environmental justice issues in marginalized neighborhoods.

Neighborhoods with the lowest vehicle ownership are, however, disproportionately located near ports, industries, and airports (e.g., first-third deciles in Figure S6a-c), and this colocation requires further investigation. Railroads (7), though, show a strong relationship with demographic variables. For example, urban neighborhoods with the lowest income have ~ 8 times more nearby railroads than neighborhoods with the highest income, and the least white urban neighborhoods have ~ 2.5 times more nearby railroads than the most white neighborhoods. Thus, of the major contributors to NO_x emissions we have examined, only roadways and railways are primarily located in marginalized urban neighborhoods (Figures S6d and 3 in the main text).

An analysis of how different emitting sectors contribute to the total urban NO_x budget from the National Emissions Inventory (NEI) (Figure S7) and how these sectors were affected by the COVID-19 lockdowns (Figure S8) sheds further light on the source of the NO_2 disparities. Heavy-duty diesel traffic (10); commercial marine activity (11); rail activity (12); and residential, commercial/institutional, and industry fuel combustion emissions (9) remained relatively unchanged during the pandemic on a nationwide basis. In contrast, light-duty traffic (10) and aviation activity (13) show sharp decreases beginning around the start of our lockdown period (Figure S8).

When taken altogether, Figures S6-S8 and Figure 3 in the main text point to passenger vehicles as the primary culprit in explaining lockdown-related NO_2 drops. Only this NO_2 source simultaneously represents a large portion of total urban NO_x emissions (Figure S7), underwent drastic decreases during the pandemic (Figure S8), and is primarily located in marginalized areas (Figure 3 in the main text). Heavy-duty trucking uses the same major highways and interstates predominately located in marginalized neighborhoods and was less affected by the pandemic on a nationwide basis (Figure S8). While emissions from aviation declined during the pandemic (Figure S8), airports are generally not located in marginalized urban neighborhoods (Figure S6c) and do not represent a significant portion of total urban NO_x emissions (Figure S7). NO_x emissions from railways (14) are generally $< 5\%$ of total urban NO_x emissions despite the collocation of railroads in marginalized neighborhoods (Figures S6d, S7). We emphasize that while other sectors beyond passenger vehicle traffic are unlikely to have played a major role in explaining the lockdown-related NO_2 drops for our nationwide analysis, within individual communities railways, ports, airports, and industry could be an important source of NO_2 disparities.

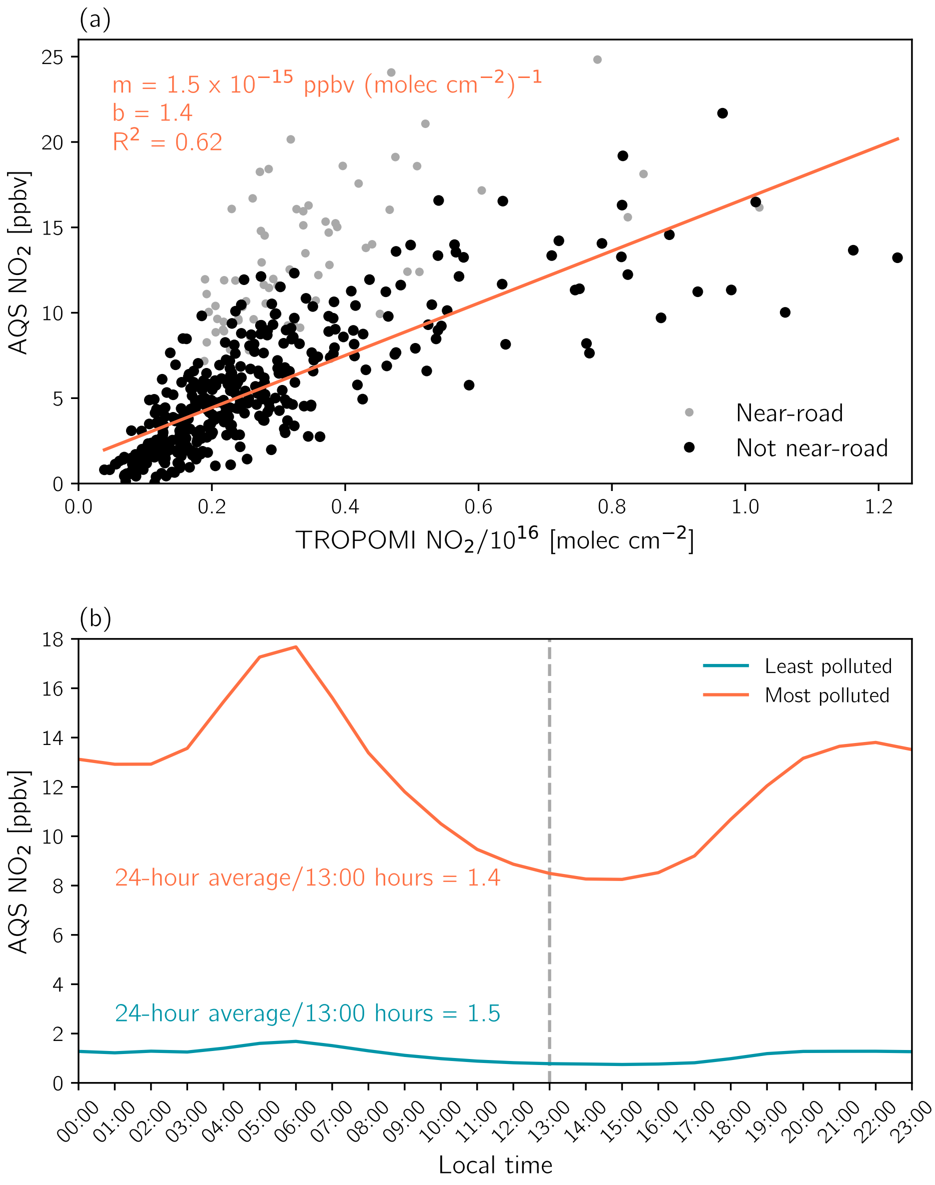

Fig. S1. (a) Observed NO_2 from AQS monitors versus TROPOMI tropospheric NO_2 columns averaged over the baseline period (13 March - 13 June 2019). TROPOMI data correspond to the nearest 0.01° latitude $\times$ 0.01° longitude grid cell to each AQS monitor. The orange line represents the linear regression fitted only through AQS data not flagged as "near-road" (< 20 meters). The orange text gives the slope (m) and intercept (b) of this linear fit. (b) Observed diurnal cycles of NO_2 averaged over the most polluted (AQS monitors where the collocated TROPOMI grid cell $>$ 90th percentile) and least polluted sites (AQS monitors where the collocated TROPOMI grid cell $<$ 10th percentile) during the baseline period. Only sites that are not near-road are considered for these averages. The ratios of 24-hour average NO_2 to NO_2 at the approximate time of satellite overpass (dashed grey line; $\sim$ 13:00 hours local time) are indicated in the colored text.

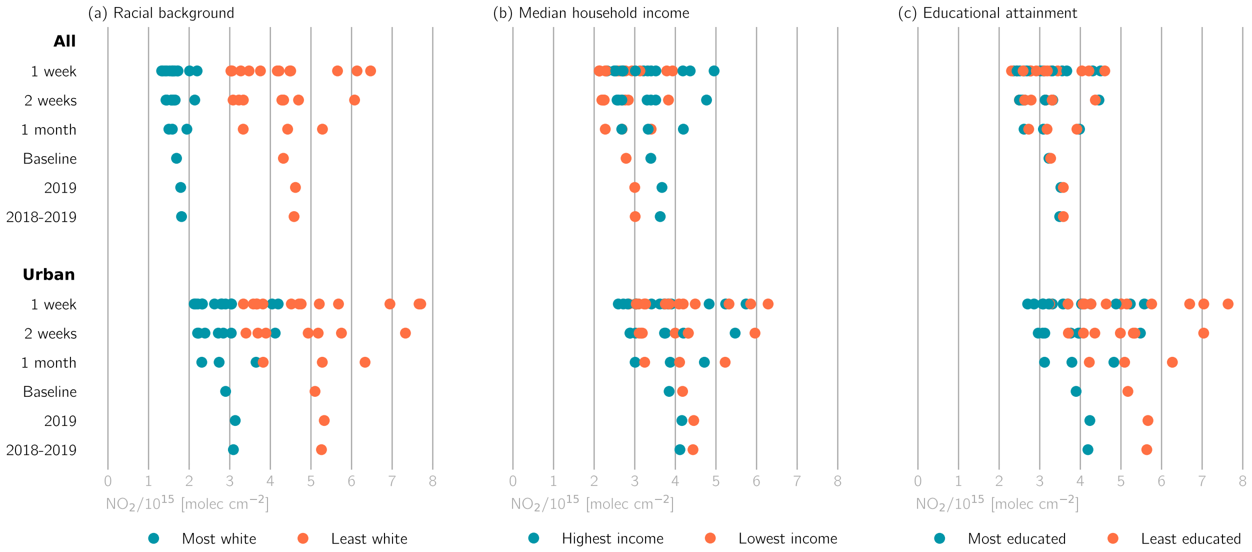

Fig. S2. Impact of meteorological variability on NO_2 disparities. Disparities are calculated similarly to Figure 2 in the main text but using the 13 one-week periods, 7 two-week periods, and 3 month-long periods that comprise the three-month baseline period used elsewhere in the study. Additionally, we show disparities calculated with NO_2 averaged over 1 January 2019 - 31 December 2019 (“2019”) and 1 May 2018 - 31 December 2019 (“2018-2019”) to illustrate their similarities to disparities found using the baseline period.

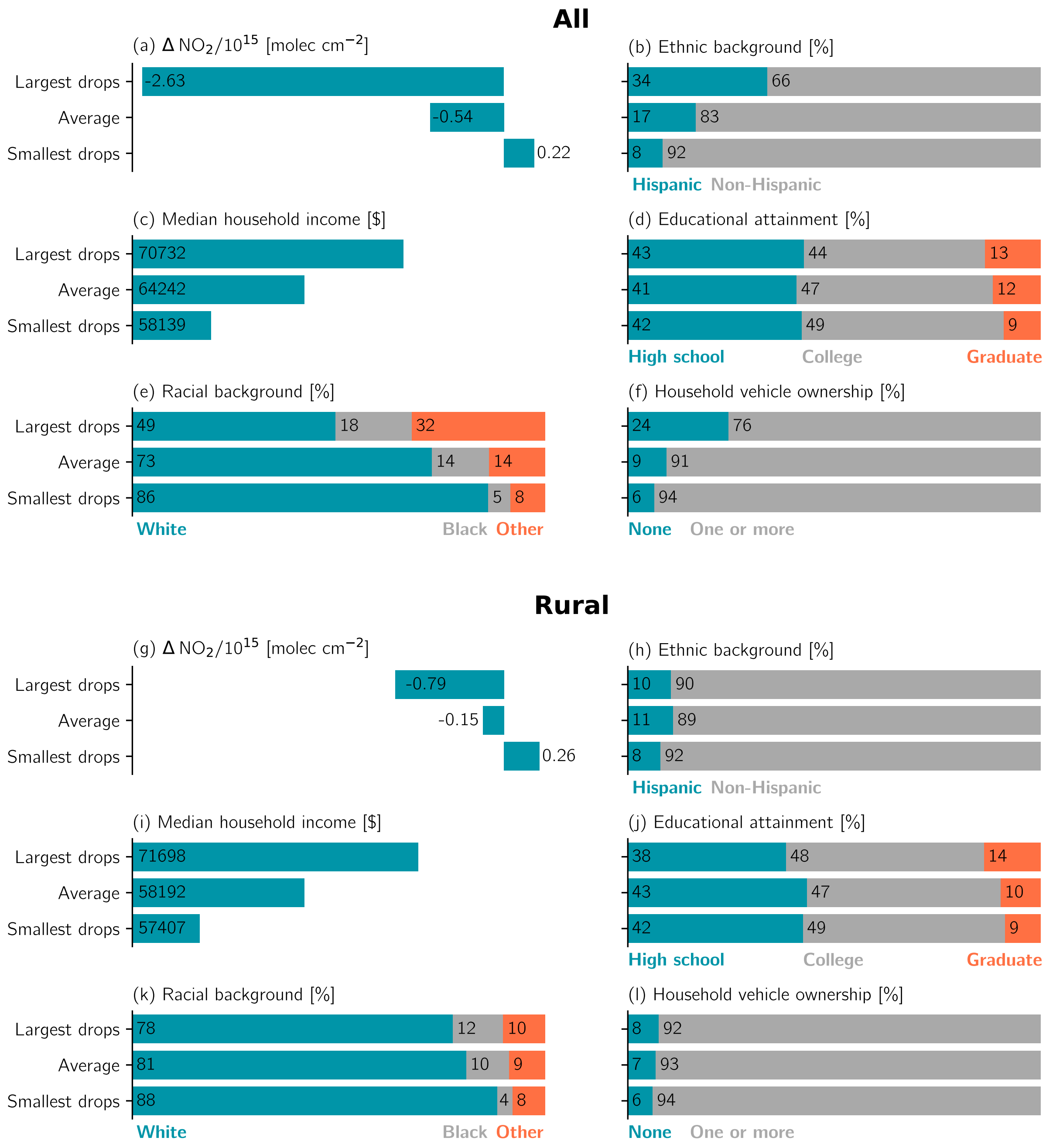

Fig. S3. Same as Figure 1c-h in the main text but drops and averages are derived from (a-f) all tracts and (g-l) rural tracts.

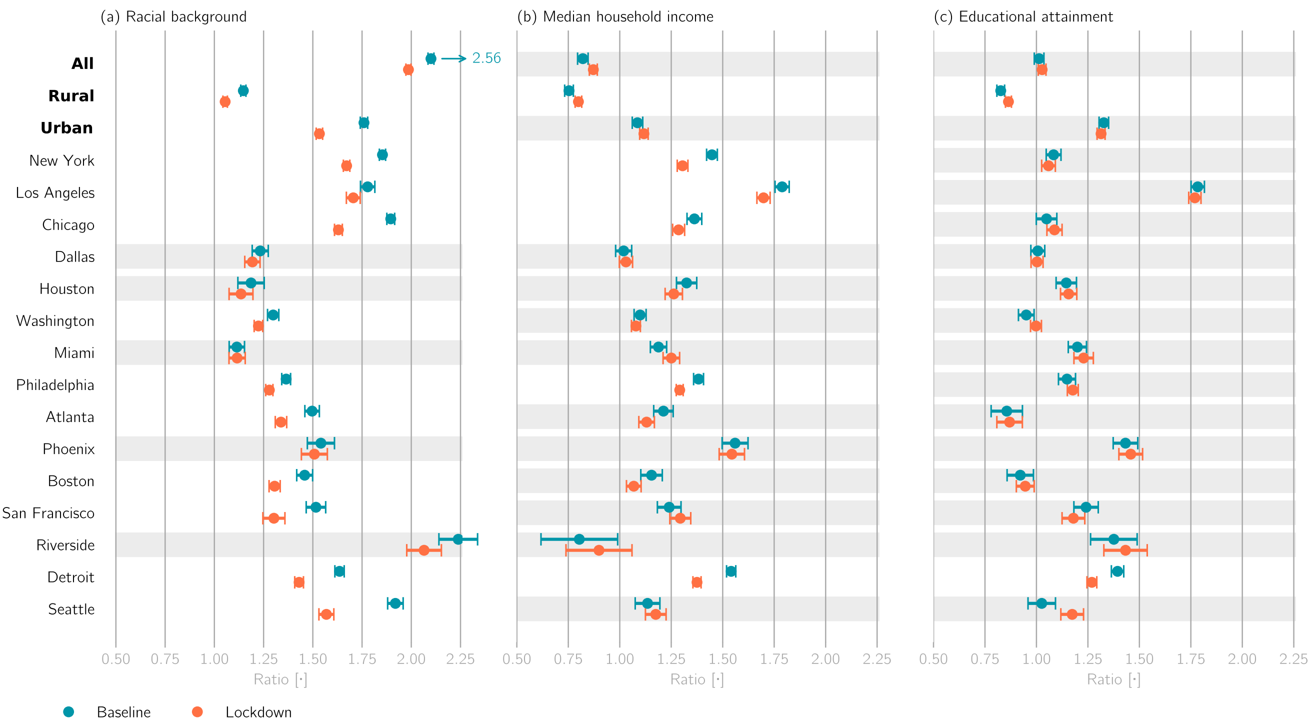

Fig. S4. Following Figure 2 in the main text, we calculate the ratio of mean NO_2 in the (a) least white to most white, (b) lowest income to highest income, and (c) least educated to most educated census tracts. Horizontal bars indicate the 95% confidence intervals for the mean ratios. Spatial conglomerations or MSAs with confidence intervals that overlap between the baseline and lockdown periods are shaded in grey.

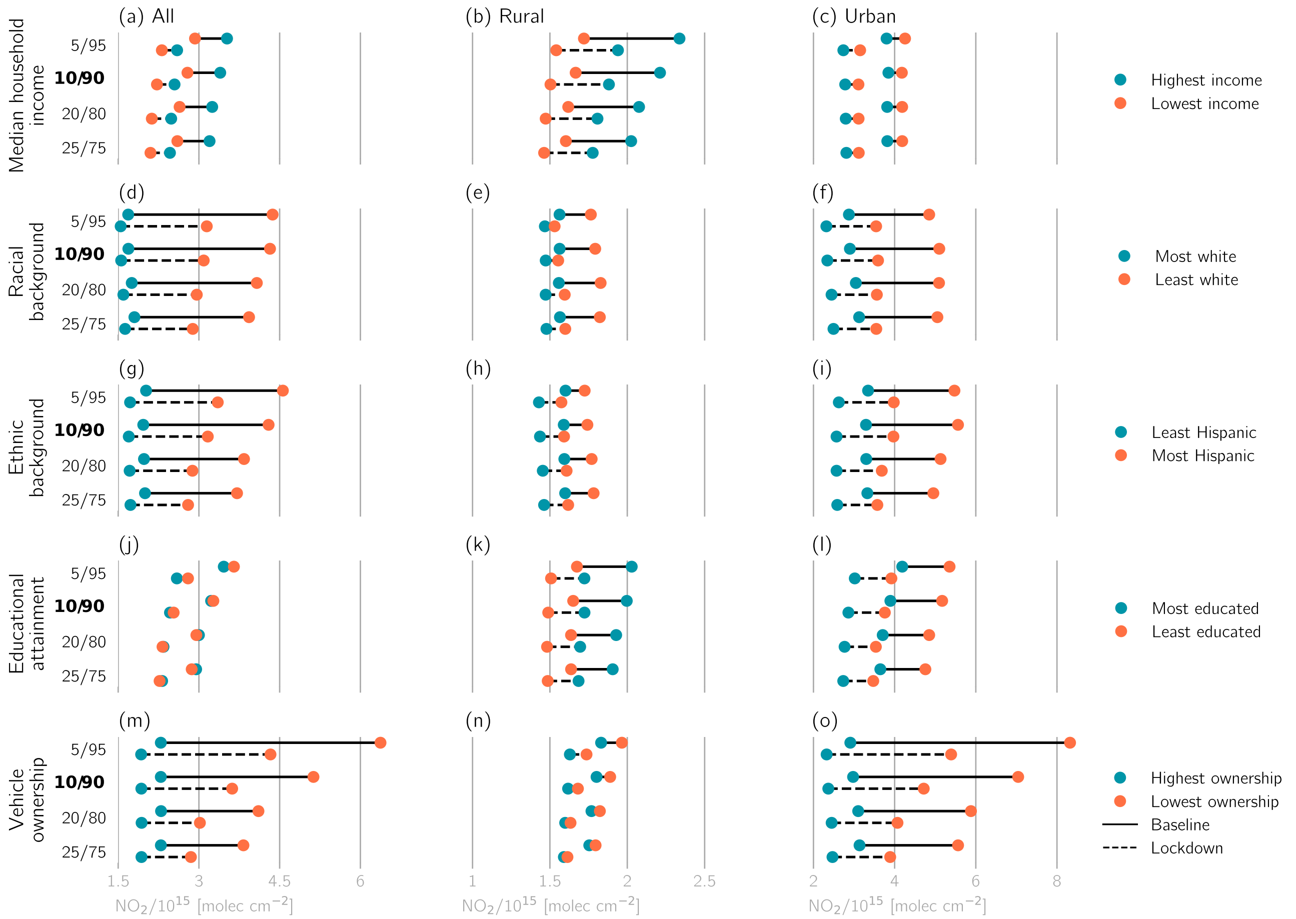

Fig. S5. Sensitivity of NO_2 disparities to percentiles chosen to constitute extreme values for each demographic variable. Interpretation follows Figure 2 in the main text, but each pair of bars in individual subplots represents different percentile thresholds, indicated in the subplots' vertical axes. The boldface 10/90 row corresponds to the first and tenth deciles used in the main text.

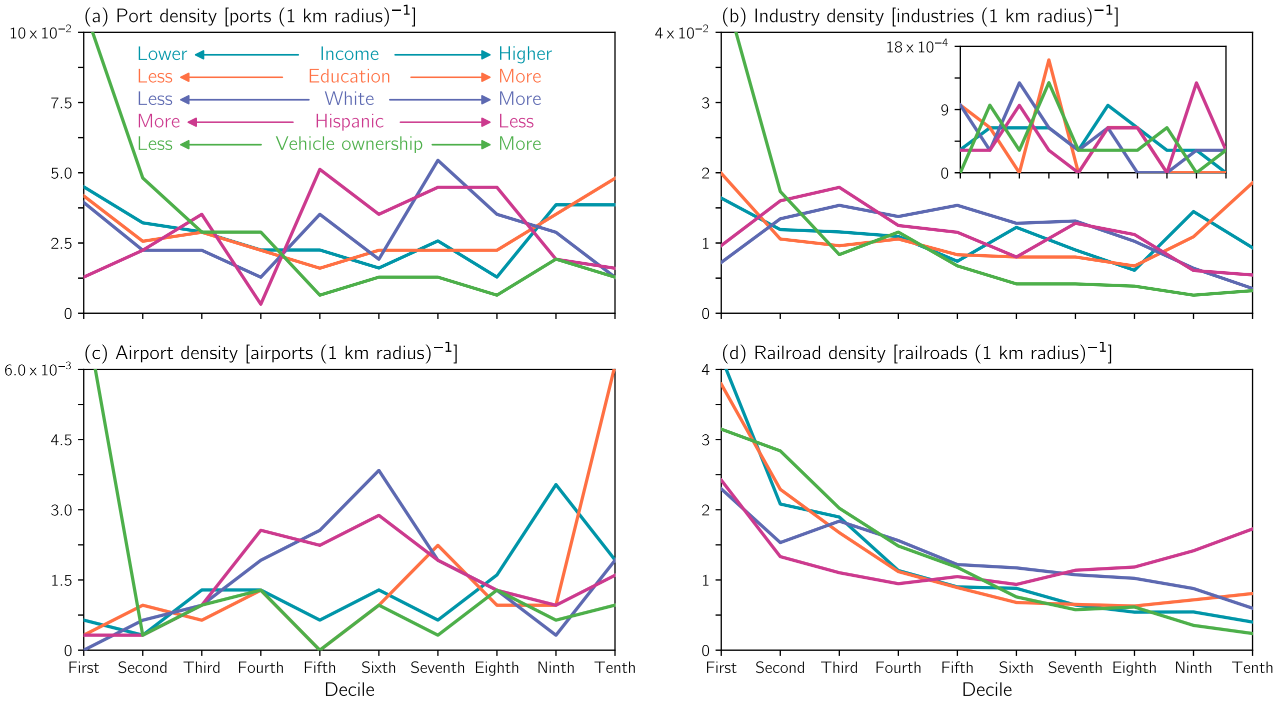

Fig. S6. The relationship of NO_2 sources with demographic variables in urban areas. Interpretation follows Figure 3 in the main text, and the color text legend in (a) applies to (b)-(d). Stationary sources outfitted with continuous emissions monitors are used as a proxy for industry in (b). The inset axis in (b) shows the same quantity as the parent axis but only for stationary sources whose cumulative NO_x emissions for 2019 exceed the 90th percentile

.

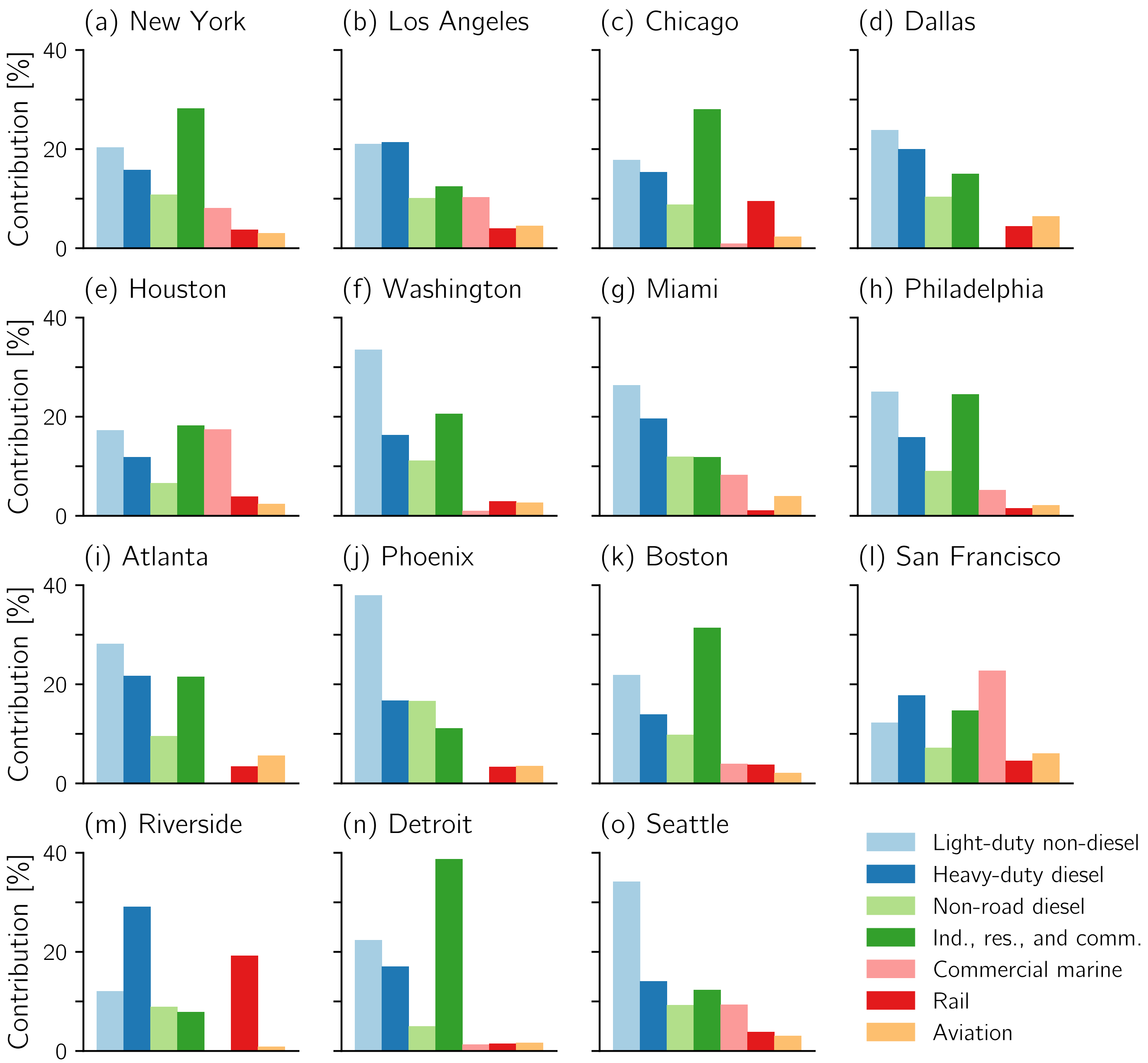

Fig. S7. NEI NO_x emissions from major contributors to the NO_x budget in the 15 largest U.S. MSAs. Note that "Res., comm., and ind." represents the sum of the NEI-designated residential, commercial/institutional, and industry fuel combustion emissions.

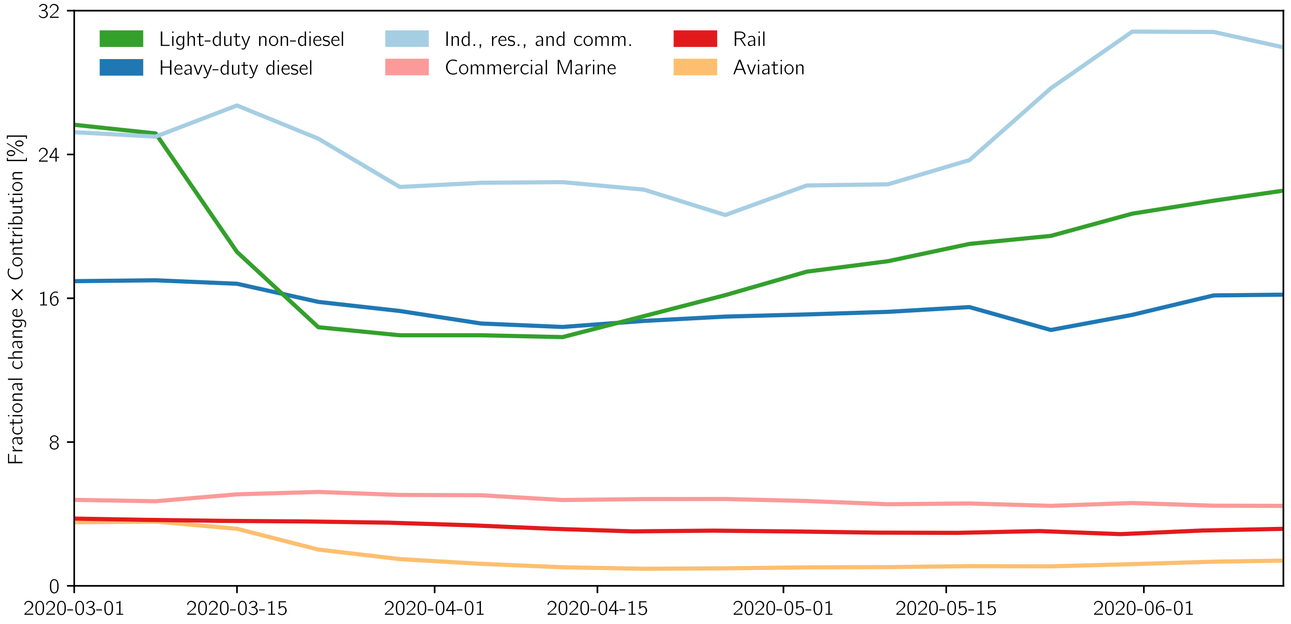

Fig. S8. The fractional change for leading contributors to urban NO_x emissions relative to 1 March 2020 values. All time series represent nationwide averages or sums. The fractional change of each contributor has been multiplied by their contribution to total urban NO_x from the NEI (e.g., heavy-duty diesel emissions represent ∼17% of the urban NO_x budget, and the fractional change time series has been multiplied by ∼17%).

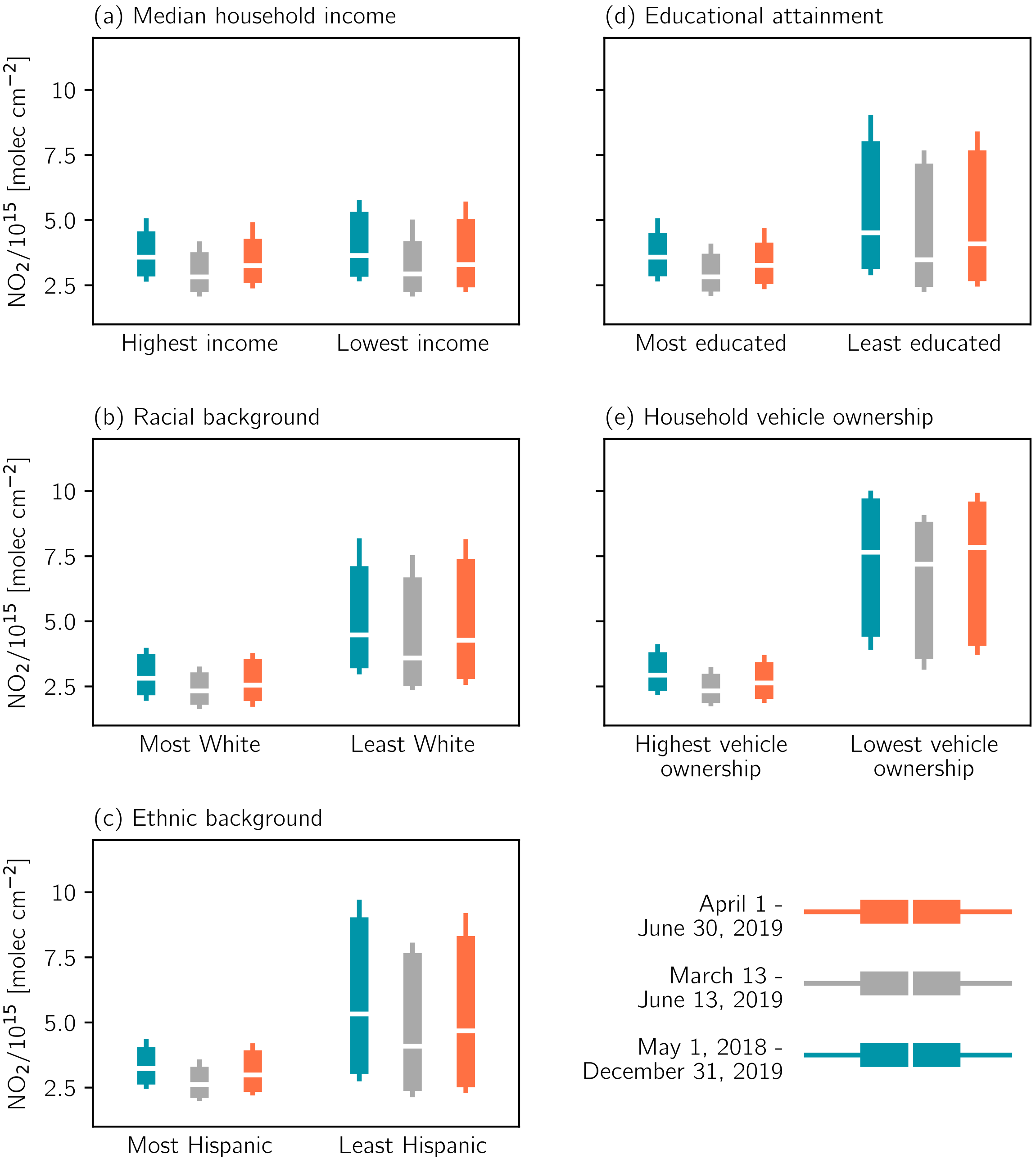

Fig. S9. Sensitivity of urban NO_2 disparities to the baseline period. Extreme values of each demographic variable (using the first and tenth deciles) for three different baseline periods: 1 April - 30 June 2019, 13 March - 13 June 2019 (the period used in the main text), and 1 May 2018 - 31 December 2019 (the entire TROPOMI data record). Boxes extend to the lower and upper quartiles of the data, and the median value is indicated with the horizontal white lines. The lower and upper whiskers extend to the 20th and 80th percentiles, respectively.

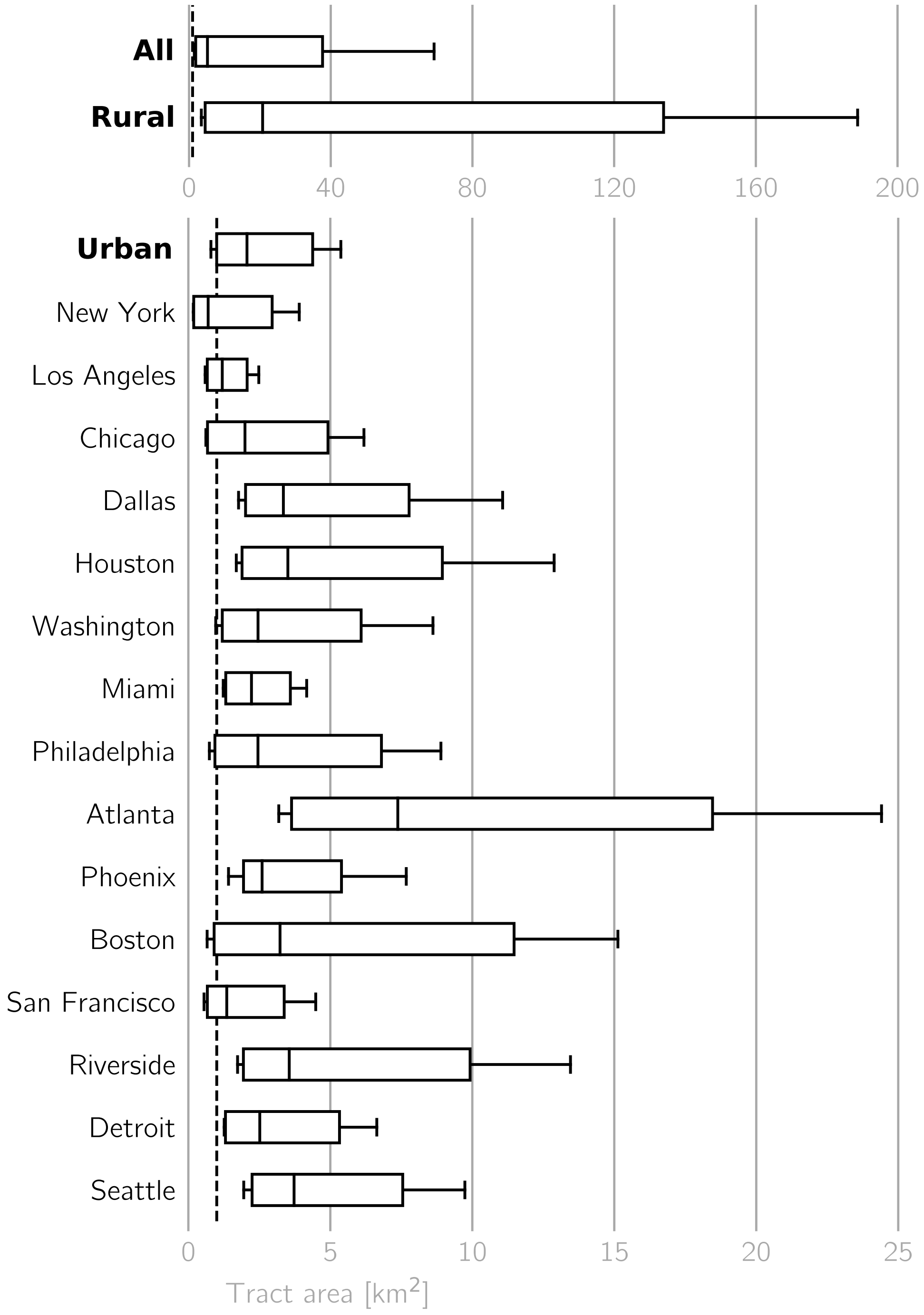

Fig. S10. Distribution of census tract areas for all, rural, and urban tracts and tracts comprising the 15 largest MSAs. From left to right in each boxplot, box lines denote the first quartile, median, and third quartile. Whiskers extend to the 20th and 80th percentiles. The dashed black line is drawn at 1 km^2, which is comparable to the area of the oversampled TROPOMI grid cells.

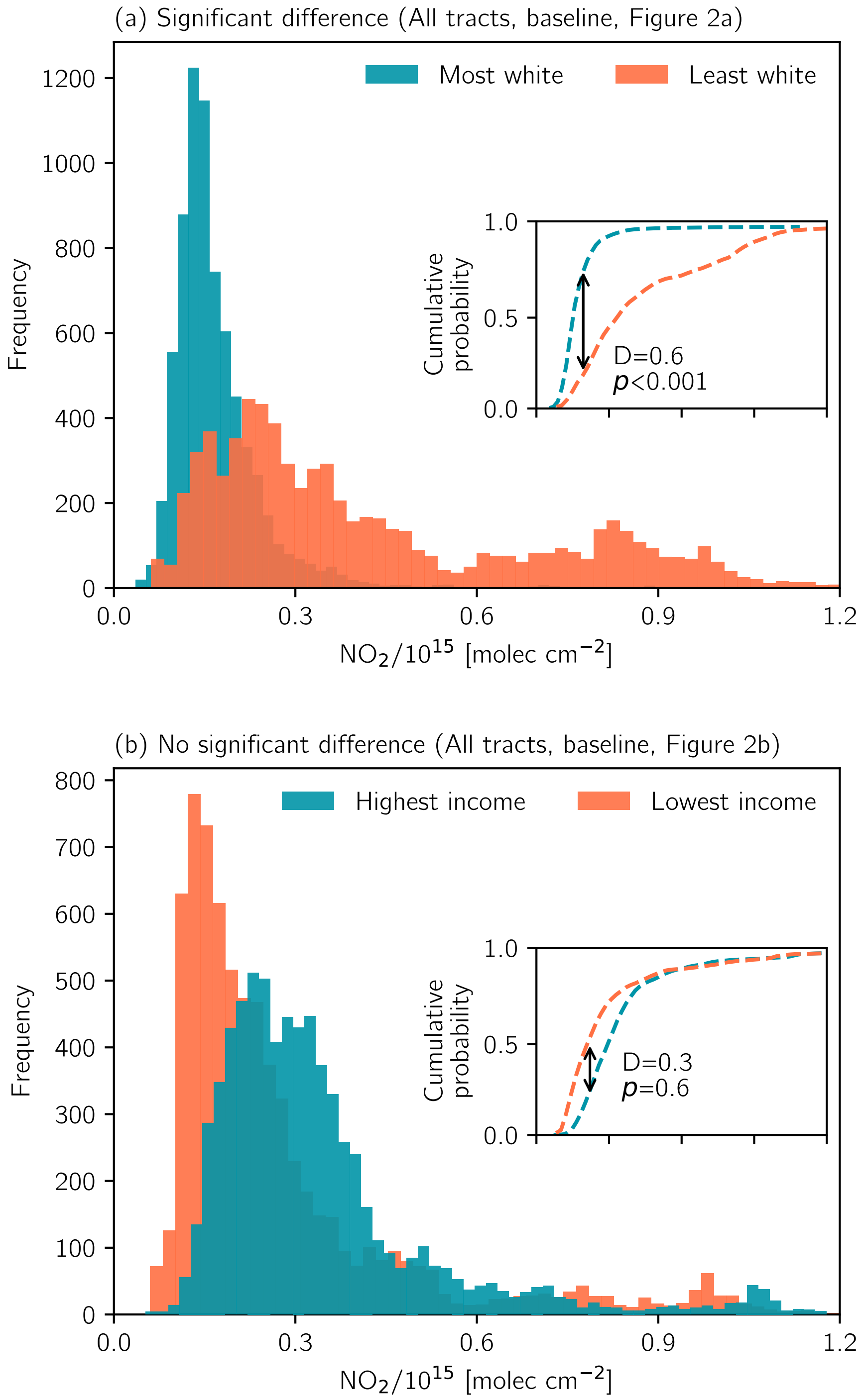

Fig. S11. Illustration of the two-sample Kolmogorov-Smirnov (KS) test used to compare whether NO_2 or demographic distributions from different population subgroups are drawn from the same distribution. NO_2 distributions are shown for (a) the most and least white census tracts and (b) the highest and lowest income census tracts (for both urban and rural tracts) during the baseline period. Inset axes in (a)-(b) illustrate the empirical cumulative distribution functions (ECDFs) for each population subgroups' NO_2 distribution. The KS test statistic, D, representing the absolute maximum distance between the ECDFs of the two distributions and the associated p values are also indicated in the inset axes. Ticks on the x-axis of the insets are identical to the parent axes. The p-value in (a) indicates that the two population subgroups with statistically different NO_2 distributions, while the large p-value in (b) indicates the difference between the two distributions is not significant at the 95% confidence level.

References

1. US Environmental Protection Agency, Air Quality System Data Mart (https://www.epa.gov/airdata) (n.d.) Accessed October 21, 2020.
2. US Environmental Protection Agency, Near-road NO_2 monitoring (https://www3.epa.gov/ttnamti1/nearroad.html) (n.d.) Accessed October 22, 2020.
3. JA Geddes, JG Murphy, JM O'Brien, EA Celarier, Biases in long-term NO_2 averages inferred from satellite observations due to cloud selection criteria. *Remote. Sens. Environ.* **124**, 210–216 (2012).
4. MJ Bechle, DB Millet, JD Marshall, Remote sensing of exposure to NO_2: Satellite versus ground-based measurement in a large urban area. *Atmospheric Environ.* **69**, 345–353 (2013).
5. LM Judd, et al., Evaluating the impact of spatial resolution on tropospheric NO_2 column comparisons within urban areas using high-resolution airborne data. *Atmospheric Meas. Tech.* **12**, 6091–6111 (2019).
6. World Food Programme, World Food Programme Global Ports database (https://geonode.wfp.org/layers/esri_gn:geonode:wld_trs_ports_wfp) (n.d.) Accessed February 16, 2021.
7. U.S. Census Bureau, TIGER/Line shapefile, 2019, nation, U.S., rails national shapefile (https://catalog.data.gov/dataset/tiger-line-shapefile-2019-nation-u-s-rails-national-shapefile) (2021) Accessed February 16, 2021.
8. OurAirports, Airports in United States of America (https://data.humdata.org/dataset/ourairports-usa) (n.d.) Accessed February 16, 2021.
9. United States Environmental Protection Agency (EPA), Air Markets Program Data (https://ampd.epa.gov) (2021) Accessed February 16, 2021.
10. Bureau of Transportation Statistics, Daily vehicle travel during the COVID-19 public health emergency (https://www.bts.gov/covid-19/daily-vehicle-travel) (2021) Accessed February 14, 2021.
11. United Nations, United Nations Comtrade Database; Global/Regional Port Calls (https://public.tableau.com/profile/uncomtrade#!/vizhome/AISPortCalls2/AISMonitor) (2021) Accessed February 18, 2021.
12. Association of American Railroads, US carloads rail traffic (https://ycharts.com/indicators/us_carloads_weekly_rail_traffic/) (2021) Accessed February 17, 2021.
13. Bureau of Transportation Statistics, The Week In Transportation; Selected transportation measures compiled by BTS during the COVID-19 public health emergency (https://www.bts.gov/covid-19/week-in-transportation#aviation) (2021) Accessed February 16, 2021.
14. United States Environmental Protection Agency (EPA), 2017 National Emissions Inventory (NEI) data (https://www.epa.gov/air-emissions-inventories/2017-national-emissions-inventory-nei-data) (2020) Accessed February 16, 2021.
